# Fractional 694‐Nm Ruby Laser Therapy for Melasma: A Clinical Study in East Asian Patients

**DOI:** 10.1111/jocd.71071

**Published:** 2026-07-16

**Authors:** Ilia Tetin, Chih‐Yu Wang, Alessandra Zevini, Daniela Martinelli, Riccardo Barini, Sherry Yunshan OuYang, Pei‐Ling Chih, Patrick Po‐Han Huang

**Affiliations:** ^1^ I‐Shou University, International College Kaohsiung Taiwan; ^2^ Department of Biomedical Engineering I‐Shou University Kaohsiung Taiwan; ^3^ El.En. Group Calenzano Italy; ^4^ Huang PH Dermatology and Aesthetics Kaohsiung Taiwan

**Keywords:** fractional laser, melasma, Q‐switched ruby laser (694 nm)

## Abstract

**Background:**

Melasma is a chronic, relapsing pigmentary disorder with a significant psychosocial impact, particularly in darker skin types. Despite various therapeutic options, its resistance to treatment necessitates optimized laser protocols and more precise evaluation tools.

**Aims:**

This study follows a dual objective: to evaluate the efficacy and safety of a low‐fluence fractional 694‐nm Q‐switched Ruby laser (QSRL) protocol in Asian patients, and to validate a novel AI‐assisted region‐of‐interest (ROI) chromatic analysis for objective clinical assessment.

**Methods:**

A retrospective analysis was conducted on patients (Fitzpatrick III–V) treated with at least four treatments using a 694 nm (QSRL) at 3–8 weeks intervals (0.77 and 0.94 J/cm^2^). Clinical outcomes were assessed using a digital imaging framework incorporating advanced feature‐alignment algorithms (SURF and RANSAC) for precise longitudinal ROI tracking. Objective improvement was quantified via Gray‐Level Co‐occurrence Matrix (GLCM) texture analysis and luminosity coefficients.

**Results:**

Sixteen patients completed the study. Significant reductions in hyperpigmentation were recorded in midfacial regions. The AI‐assisted analysis demonstrated high sensitivity in capturing localized improvements and textural shifts often missed by global clinical impressions. Notably, no cases of PIH or rebound pigmentation occurred during the 1‐year follow‐up. High patient satisfaction correlated strongly with quantitative pigmentary transitions.

**Conclusion:**

Low‐fluence fractional 694‐nm QSRL provides a safe, effective treatment option for melasma in Asian skin. Moreover the study validates the AI‐assisted ROI analysis provides a superior assessment tool. This dual approach offers a promising paradigm for long‐term melasma management, combining an optimized laser protocol with a robust, reproducible benchmark for precision dermatology.

**Abbreviations**
MTZ: microscopic thermal zones
PIH: Post‐Inflammatory Hyperpigmentation
ROI: Region of Interest

## 1 Introduction

Melasma is a chronic pigmentary disorder characterized by symmetric hyperpigmented patches on the face, predominantly affecting women and individuals with darker skin tones (Fitzpatrick types III‐VI) [1]. Beyond its clinical presentation, its relapsing nature exerts a profound psychosocial impact, significantly impairing self‐esteem and quality of life. The pathogenesis is complex and multifactorial, involving an interplay of UV radiation, hormonal shifts, and chronic inflammation linked to photoaging, characterized by increased vascularity and the activation of pro‐inflammatory mediators. Despite various therapeutic approaches focusing on sun protection and topical agents, its resistance to treatment poses ongoing challenges [2].

Laser‐based therapies have emerged as a promising alternative, yet the use of traditional, high‐fluence lasers in darker skin types has historically been limited by the significant risk of post‐inflammatory hyperpigmentation (PIH).

Recently, the development of low‐fluence, fractional Q‐switched laser technologies has shifted the treatment paradigm. These methods deliver energy in a pixelated pattern, creating micro‐zones of damage that promote faster healing and minimize downtime. Specifically, the 694‐nm Q‐switched Ruby Laser (QSRL) may offer a unique therapeutic window due to its high selectivity for melanin and minimal absorption by competing chromophores, effectively minimizing thermal damage [3, 4, 5].

However, evaluating the efficacy of these technological advancements remains a significant hurdle. Current “gold standard” assessment tools, such as the Melasma Area and Severity Index (MASI), introduced in 1994 by Kimbrough‐Green et al. [6], rely on global visual impressions and are prone to inter‐observer variability. These traditional methods fail to capture localized chromatic shifts or subtle changes in spatial distribution, and they often overlook the crucial contrast between lesional and adjacent normal skin. Because clinical response to laser therapy is often region‐specific and heterogeneous, conventional scales may even fail to detect paradoxical aggravation or lesion enlargement.

The quest for more sensitive and objective assessment tools in melasma has led to the proposal of several alternatives to the traditional MASI score. Recent efforts have focused on enhancing the weight of pigment intensity, as seen in the Melasma Severity Index (MSI), which utilizes a squared parameter for darkness to better reflect subtle chromatic improvements [7].

Other researchers have explored quasi‐quantitative methods such as the Point Counting‐Serial Image Index (PCSI) [5], designed to track the spatial fragmentation of lesions, a common occurrence following fractional laser therapy that global scales often fail to quantify [8]. Furthermore, the push toward Digital Image Analysis (DIA), utilizing metrics like the Weighted Skin Intensity (WSI) ratio and the L*a*b* color space, aims to eliminate inter‐observer subjectivity by providing a purely mathematical evaluation of the contrast between lesional and perilesional skin.

Despite these advancements, most of these emerging scales are still undergoing validation or require specialized equipment. Consequently, the present study is designed with a dual objective: first, to evaluate the clinical efficacy of a specific low‐fluence fractional 694 nm QSRL protocol in Asian patients (skin Type III, IV and V); and second to validate a more adequate AI‐assisted quantitative imaging framework for monitoring such improvements.

By employing a two‐stage alignment process based on SURF‐feature [9] alignment and RANSAC [10], we ensured that identical midfacial Regions of Interest (ROIs) were analyzed with high reproducibility. Unlike methods that rely solely on global visual impressions, our approach integrates Gray‐Level Co‐occurrence Matrix (GLCM) texture analysis and luminosity coefficients to track the evolution of both pigment intensity and textural uniformity. This dual‐purpose methodology not only assesses the therapeutic performance of the ruby laser but also addresses the diagnostic gap in melasma, offering a granular, objective benchmark that captures treatment‐induced changes often missed by traditional scoring systems.

## 2 Methods

### 2.1 Study Setting

This study is based on a real‐world data analysis, utilizing data from a retrospective chart review from the database of theHuang PH Dermatology and Aesthetics, Kaohsiung, Taiwan. Data were collected from May 2022 to May 2023.

The analysis encompasses patients who were diagnosed with mixed melasma, persisting for over 10 years, who had not undergone laser treatment or used topical bleaching agents within three months prior to the initial treatment. All included patients received at least four treatment sessions, spaced 3–8 weeks apart, using the solid‐state fractionated 694 nm ruby laser (Q Plus R, Quanta System S.p.A., Samarate, Italy), up to max five sessions.

Patients were excluded if they presented with any of the following: concomitant use of photosensitizing agents, retinoids, isotretinoin, anticoagulants or immunosuppressants, a history of epilepsy or suspected epileptic reactions during treatment; pregnancy or breastfeeding women; presence of cancerous or precancerous lesions, Herpes simplex, or wounded skin in the treatment areas; high skin tanning.

**FIGURE 1:**
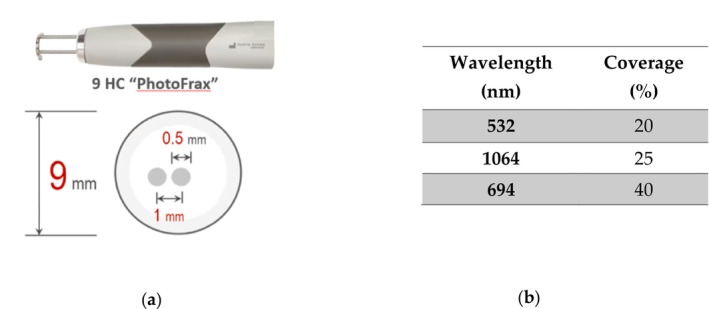
Representative image of 9 mm High Coverage handpiece (9 HC).

The treatment was performed with 694 nm ruby laser at fractional 9 mm High Coverage (9 HC) handpiece (Figure 1). The parameters used were fluence of 0.77 to 0.9 J/cm^2^, Q‐Switched pulse duration of 6 ns. Each patient was scanned with non‐overlapped sweeping.

**FIGURE 2:**
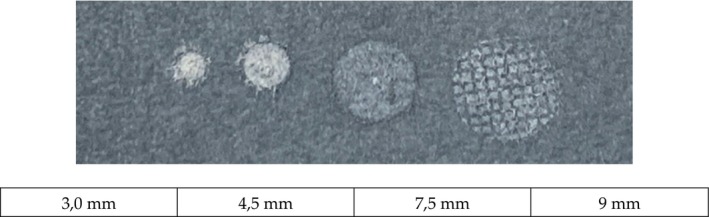
Representative image of 9 mm High Coverage handpiece (9 HC) energy distribution.

The laser energy is delivered with a fractionated handpiece to control collateral thermal damage of the skin tissue when longer pulse duration is used. The scanner delivers each energy pulse in a round of 63,62 mm^2^ area and fractionalizes the energy in circular dots of 0,5 mm in diameter with a pitch of 1 mm for the 694 nm Ruby laser the coverage is up to 40% of 63,62 mm^2^ (Figure 1). Contributing to a good uniformity of energy distribution, representing a good compromise compared to previously developed versions or spot sizes (Figure 2). The endpoints were mild redness, which quickly faded in a few minutes.

### 2.2 Patient Management

Topical anesthesia with lidocaine‐prilocaine (EMLA) applied to the whole face under occlusion for 1 h. The anesthetic cream was removed immediately before laser surgery using wet gauze.

Oral tranexamic acid was not prescribed to any of the 18 included patients.

### 2.3 Clinical Evaluation

Objective improvement parameters were assessed via clinical examination and standardized digital photography. Image acquisition was performed using a 2D digital imaging camera (Nikon D5200, Tokyo, Japan) under controlled lighting and standardized geometric registration.

These images served as the primary data source for a quantitative, automated evaluation system designed to analyze the intensity of pigmentation within a specific region of interest (ROI), located at the darkest area of the cheekbones.

The intensity of pigmentation was quantified by categorizing the skin within the ROI into three specific phototypes (III‐V), representing the most prevalent tones in the Asian population [11].

Although the Fitzpatrick system is formally defined by skin response to ultraviolet exposure (burning or tanning), in clinical practice dermatologists usually infer these responses directly from baseline skin color, which is more practical and widely understood. Therefore, a representative phototype III skin tone was first selected as the reference baseline. Darker pigmentation patterns observed within melasma lesions were subsequently categorized as Type IV and Type V, with Type V operationally defined as the darkest homogeneous pigmentation observed within the lesion area.

During image analysis, representative regions corresponding to these three pigmentation types were used to establish thresholds based on color and lighting proxies derived from digital image channels. Type III generally corresponded to lighter brown areas with higher luminance, Type IV to intermediate brown pigmentation, and Type V to darker, more homogeneous brown regions.

Pigmentation intensity was defined quantitatively from the registered region of interest (ROI) as a measure of skin darkness, computed from standardized color values under controlled imaging conditions. This was derived from the Value (V) channel in HSV and luminance‐weighted brightness in RGB, where lower V or lower luminance indicates higher pigmentation intensity. Reference samples were established through camera‐calibrated, within‐image color anchoring rather than comparisons across ethnic groups. In this study, the clinically recognized skin tones were translated into quantifiable image‐derived signals so that pigmentation classification could be objectively identified from optical properties within a fixed cheekbone ROI.

Under standardized acquisition and geometric registration, pigmentation intensity was calculated from the ROI's lightness/luminance after masking invalid pixels. Severity was quantified using two complementary metrics: (1) a continuous darkness measure (ΔV or Δluminance relative to baseline) and (2) an area‐based metric representing the proportion of the ROI assigned to darker calibrated bins (Types III–V), where a higher Type V fraction indicates greater pigmentation burden. The ROI was positioned at the darkest cheekbone region so that measurements reflected lesion‐dominant pigmentation rather than overall facial skin tone.

For pre‐ and post‐treatment analysis, follow‐up images were acquired using a digital imaging system (VISIA, CANFIELD Imaging System, Fairfield, NJ, USA).

This data, as well as all other medical information, was collected in full compliance with ethical standards: all patients signed photographs for future research purposes. The management of this data was carried out in accordance with institutional guidelines, and the study received approval from the Mackay Memorial Hospital Institutional Review Board (IRB), Approval certificate for the clinical trial “Application of AI‐assisted analysis and diagnostic system for pigmentary skin disorders.” IRB No. 21MMHIS317e. Taipei (Taiwan): Mackay Memorial Hospital; 2022 May 19.

### 2.4 Skin Color Measuring Device

The automatic system processes standardized facial photographs taken under consistent lighting conditions using a 2D camera (Nikon D5200, Tokyo, Japan). Images are captured at a resolution of 4928 × 3264 pixels. All photographs are taken perpendicular to the facial surface, with subjects positioned using a standardized chin rest and forehead support to maintain consistent alignment between sessions.

The system aligns facial images across sessions via a two‐stage approach. First, an operator defines a baseline ROI, selecting an area of at least 100 × 100 pixels. In the second stage, a SURF‐based feature alignment [9] automatically aligns follow‐up ROIs using a holography matrix estimated with Lowe's ratio test [12] and RANSAC [10]. This process robustly maps points from the follow‐up image to the baseline image by minimizing the effect of outliers, ensuring that identical skin areas are analyzed over time and enhancing the reliability of our results.

Quality metrics ($Q_{1}$, $Q_{2}$, $Q_{3}$)are then calculated across non‐overlapping 100 × 100 pixel windows in the ROI containing at least 80% valid skin pixels. Pixels are considered valid if their color values fall within the typical range of human skin tones and are not saturated or underexposed. For $Q_{1}$, we calculate the signal‐to‐noise ratio in the HSV space:

$$Q_{1}=\frac{\eta_{H}}{SD_{H}}+\frac{\eta_{S}}{SD_{S}}+\frac{\eta_{V}}{SD_{V}},$$

where $\eta_{H},\eta_{S},\eta_{V}$, are the median values of the hue, saturation, and value channels, respectively; $SD_{H},SD_{S},SD_{V}$ are the corresponding standard deviations of these channels.

For $Q_{2}$, we use luminosity coefficients derived from the human vision luminosity model to weight the contributions of each color channel in RGB color space, emphasizing the green channel:

$$Q_{2}=0.2989\frac{\eta_{R}}{SD_{R}}+0.5870\frac{\eta_{G}}{SD_{G}}+0.1140\frac{\eta_{B}}{SD_{B}}.$$

$Q_{3}$ leverages texture analysis via Gray‐Level Co‐occurrence Matrix (GLCM) features, summarizing contrast, homogeneity, energy, and correlation [13]. Let $G\left(i,j\right)$ be the normalized GLCM, N be the number of gray levels, and $\mu_{i,},\mu_{j},\sigma_{i},\sigma_{j}$ be the means and standard deviations of the marginal distributions. Contrast $Q_{3c}$ emphasizes the difference between gray levels of neighboring pixels:

$$Q_{3c}=\sum_{i=0}^{N-1}\sum_{j=0}^{N-1}{\left(i-j\right)}^{2}G\left(i,j\right).$$

Homogeneity $Q_{3h}$ measures the closeness of the distribution of elements in the GLCM to the diagonal:

$$Q_{3h}=\sum_{i=0}^{N-1}\sum_{j=0}^{N-1}\frac{G\left(i,j\right)}{1+\left|i-j\right|}.$$

Energy $Q_{3e}$ represents the textural uniformity of the image:

$$Q_{3e}=\sum_{i=0}^{N-1}\sum_{j=0}^{N-1}{\left[G\left(i,j\right)\right]}^{2}.$$

Correlation $Q_{3r}$ evaluates how much a pixel is correlated to its neighbor in terms of gray levels:

$$Q_{3r}=\frac{\sum_{i=0}^{N-1}\sum_{j=0}^{N-1}\left(i-\mu_{i}\right)\left(j-\mu_{j}\right)G\left(i,j\right)}{\sigma_{i}\sigma_{j}}$$

**FIGURE 3:**
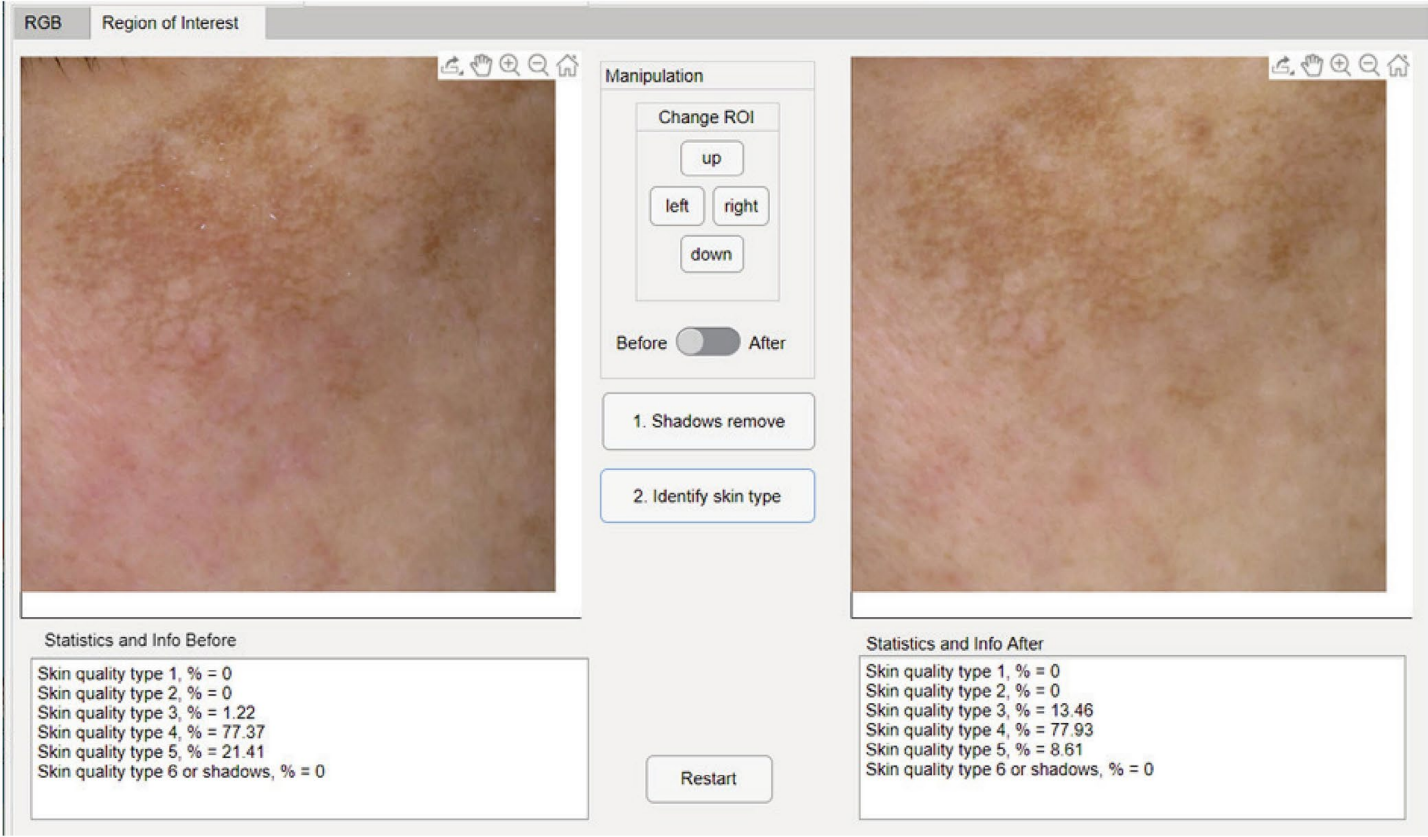
User interface of the skin color and quality measurement system, displaying the analysis of a region of interest (ROI) before and after processing. The system quantifies different skin quality types and handles shadow removal for accurate classification, as detailed in the pre and post‐processing statistics.

We combine the GLCM features into a single metric $Q_{3}$, by computing their mean. The composite metric CM, derived from $Q_{1},Q_{2}$ and $Q_{3}$ quality metrics, is compared with thresholds to provide a quantitative skin type breakdown for the ROI. Classification was calibrated using a training set of 500 expert‐labeled images, encompassing all Fitzpatrick skin types. Validation was performed on an independent test set of 100 images, achieving 93.2% accuracy and an F1 score above 0.90 Figure 3.

### 2.5 Statistical Analysis

Statistical analysis was performed using GraphPad Prism (v10.3 for Windows, GraphPad Software, www.graphpad.com). Based on clinical rationale, we hypothesized an increase in Type III and IV, and a decrease in Type V over time. A paired *t‐ test* was employed to specifically test these hypotheses and to compare the skin improvement among different treatment sessions. A *p‐value* < *0.05* was considered statistically significant.

## 3 Results

In this study, a total of 18 patients met the inclusion and exclusion criteria, while 16 patients completed the treatment session and were included in the analysis. The mean age was 49 years (range: 40–66 years). Fitzpatrick skin types were predominantly type IV (17/18 patients, 94.4%), with one patient presenting with type III skin type (5.6%) (Table 1).

**TABLE 1:** Further breakdown of melasma before treatment: skin color distribution.

Melasma	Gender M/F	Skin type III/IV	Age (mean, max, min)
Mixed‐type	0%/100%	5.6%/94.4%	49, 66, 40

All cases were clinically classified as mixed‐type hyperpigmentation, involving both epidermal and dermal components.

Pigmentation analysis was conducted on a cohort of 16 female patients, the vast majority of whom presented with Fitzpatrick skin type IV. As a result, the pigmentation assessment focused on Types III, IV, and V, given the absence of cases corresponding to other classifications.

Each patient underwent a minimum of three treatment sessions, with 11 out of 16 patients completing a fourth observation point (Obs4) and 10 out of 16 patients completing a fifth observation point (Obs5), enabling longitudinal analysis over time.

Pigmentation was classified into three distinct types based on clinical photographs: Type III, characterized by fine dots or globules (typically more superficial); Type IV, consisting of diffuse, patchy brown pigmentation; and Type V, marked by homogenous dark brown areas often associated with deeper dermal deposition.

Quantitative assessments were performed by calculating the percentage area involved for each pigmentation type, separately for the right (R) and left (L) sides of the face, as well as an overall (O) average derived from both regions. These percentages were recorded at five observation points (Obs1 to Obs5), corresponding to the sequential treatment sessions. Data are reported as mean ± standard deviation, and temporal changes were evaluated statistically using paired *t*‐test (1‐tailed), with Obs1 serving as the baseline.

This analytical approach allowed for a comprehensive evaluation of how each pigmentation subtype responded to treatment over time, with attention to spatial distribution (right, left, overall) and the persistence or resolution of pigmentary features. The results are structured into two parts: first, a detailed analysis of Types III, IV, and V individually; and second, a combined assessment comparing the overall evolution of superficial pigmentation (Type III + IV) against deeper pigmentation (Type V).

**FIGURE 4:**
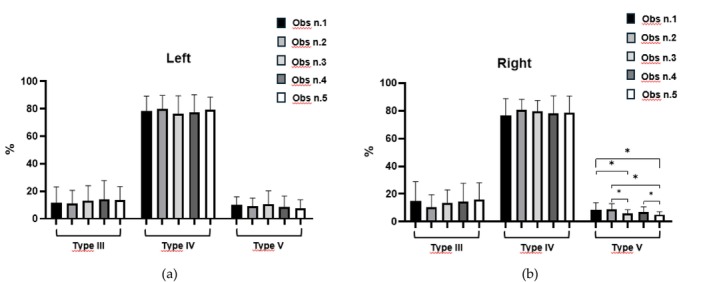
Comparative test between different sessions and different kind of pigmentation (III, IV and V). (a) Measures in the left cheek; (b) Measures of the right cheek. *Statistical significance:* *p < 0.05.

On the right side (R), for Type III pigmentation, values ranged from 10.47% to 15.99%, with no statistically significant variation over time (*t*‐test, p1vs5 = 0,172). Type IV pigmentation showed stable values across sessions (range: 76.74%–80.61%), with no significant differences (*t*‐test, p1vs5 = 0,428). Notably, Type V showed a decrease in mean pigmentation from 9.10% (Obs2) to 5.18% (Obs5), with significant differences between Obs1 and Obs5 (*t*‐test, *p* = 0,036). Significance on Type V seems evident also comparing different sessions performed on the right cheek, starting from the second session (*t*‐test, p2vsp3 = 0,044; p2vsp5 = 0,045), as indicate in Table 2 and Figure 4.

**TABLE 2:** Comparison between sessions for single side of treatment (right or left cheek) and overall (right + left cheek).

	Obs. n.1	Obs. n.2	Obs. n.3	Obs. n.4	Obs. n.5	*p* (obs. I vs. V)
**R:Type III (Mean ± SD)**	15,06 ± 13,81	10,47 ± 8,94	13,72 ± 9,22	14,61 ± 13,13	15,99 ± 11,51	0,172
**R:Type IV (Mean ± SD)**	76,74 ± 11,96	80,61 ± 7,69	79,54 ± 7,99	78,06 ± 12,73	78,87 ± 11,13	0,428
**R:TypeV (Mean ± SD)**	8,31 ± 5,40	9,10 ± 3,95	5,80 ± 2,95	7,17 ± 3,44	5,18 ± 1,90	0.036*
**L:Type III (Mean ± SD)**	11,48 ± 11,71	11,24 ± 9,45	13,10 ± 10,94	14,04 ± 13,7	13,64 ± 9,82	0,258
**L:Type IV (Mean ± SD)**	78,55 ± 10,72	79,78 ± 10,01	76,31 ± 13,10	77,23 ± 13,03	79,24 ± 9,21	0,445
**L:Type V (Mean ± SD)**	9,89 ± 6,07	9,00 ± 6,04	10,62 ± 9,72	8,73 ± 7,82	7,61 ± 6,21	0,235

* *p* < 0.05.

On the left side of the face, values for Type III ranged between 11.24% and 14.04% with no statistical significance across timepoints. Type IV values remained consistent (78.55%–79.78%), and no significant differences were detected. Type V values declined slightly (from 9.89% to 7.61%) with no significant changes (*t*‐test, p1vs5 = 0,235).

**FIGURE 5:**
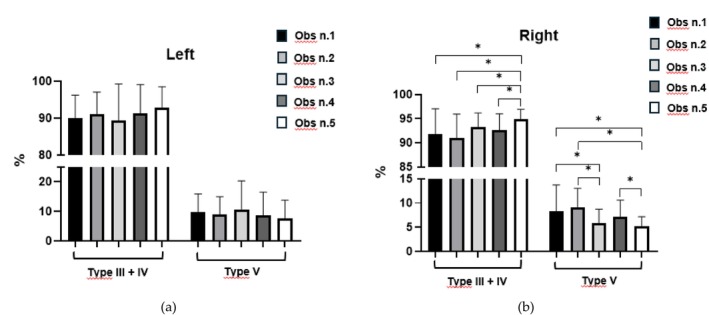
Comparative test between different sessions and different kinds of pigmentation (III + IV and V). (a) Measures in the left cheek; (b) Measures of the right cheek. Statistical significance: *p < 0.05.

On the right side of the face, for type III + IV combined values increased slightly from 91.80% (Obs1) to 94.87% (Obs5), with a significant reduction in variability (std: 5.24% to 1.96%; *t*‐test *p* = 0.024). Notably, Type V showed a decrease in mean pigmentation from 9.10% (Obs2) to 5.18% (Obs5), with significant differences between Obs1 and Obs5 (*t*‐test, *p* = 0.036). On the other hand, the left side of the face presented for Type III + IV, a gradual increase (from 90.02% to 92.87%), but this was not statistically significant (*t*‐test p1vs5 = 0.130). Type V values declined slightly (from 9.89% to 7.61%) with no significant changes (*t*‐test p1vs5 = 0.235) (Table 3 and Figure 5).

**TABLE 3:** Comparison between the session's mean for single side of treatment (right or left cheek) and overall (right + left cheek).

	Session n.1	Session n.2	Session n.3	Session n.4	Session n.5	*p* (session I vs. V)
**R:Type III + IV (Mean ± SD)**	91,80 ± 5,24	91,08 ± 4,86	93,26 ± 2,62	92,67 ± 3,37	94,87 ± 1,96	0,024*
**R:TypeV (Mean ± SD)**	8,31 ± 5,40	9,10 ± 3,95	5,80 ± 2,95	7,17 ± 3,44	5,18 ± 1,90	0,036*
**L:Type III + IV (Mean ± SD)**	90,02 ± 6,23	91,02 ± 6,04	89,41 ± 9,86	91,27 ± 7,82	92,87 ± 5,62	0,130
**L:Type V (Mean ± SD)**	9,89 ± 6,07	9,00 ± 6,04	10,62 ± 9,72	8,73 ± 7,82	7,61 ± 6,21	0,235

*Note:* The asterisk (*) indicates statistical significance (*p* < 0.05).

Overall pigmentation trends mirrored the side‐specific values. Type III + IV increased from 90.91% to 93.87% with borderline statistical significance (*t*‐testp1vs5 = 0,051).

**FIGURE 6:**
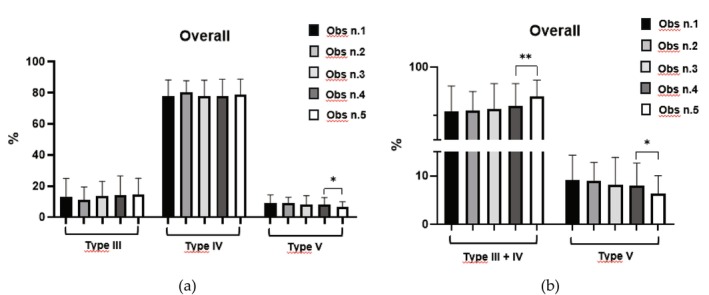
Overall **c**omparative test between different sessions and different kinds of pigmentation (a) Overall measures of III, IV, and V; (b) Overall measures of III + IV vs. V.Statistical significance: *p < 0.05; **p < 0.01.

Type V decreased from 9.10% to 6.39%, with a near‐significant *p*‐value (*t*‐test *p* = 0, 100) (Table 4 and Figure 6).

**TABLE 4:** Comparison between sessions for overall (right + left cheek).

	Session n.1	Session n.2	Session n.3	Session n.4	Session n.5	*p* (session I vs. V)
**O:Type III (Mean ± SD)**	13,27 ± 11,92	10,85 ± 8,64	10,62 ± 9,72	14,33 ± 12,24	14,81 ± 10,21	0,198
**O:Type IV (Mean ± SD)**	77,64 ± 10,86	80,20 ± 7,66	77,92 ± 10,18	77,65 ± 11,06	79,05 ± 9,76	0,427
**O:Type V (Mean ± SD)**	9,10 ± 5,35	9,05 ± 3,73	8,21 ± 5,62	7,95 ± 4,70	6,39 ± 3,65	0,100
**O:Type III + IV (Mean ± SD)**	90,91 ± 5,34	92,05 ± 3,91	91,33 ± 5,24	91,97 ± 4,62	93,87 ± 3,45	0,051

There was no rebound pigmentation, expanded melasma areas, sensitive skin, or hypopigmentation at least 6 months following the final treatment session.

**FIGURE 7:**
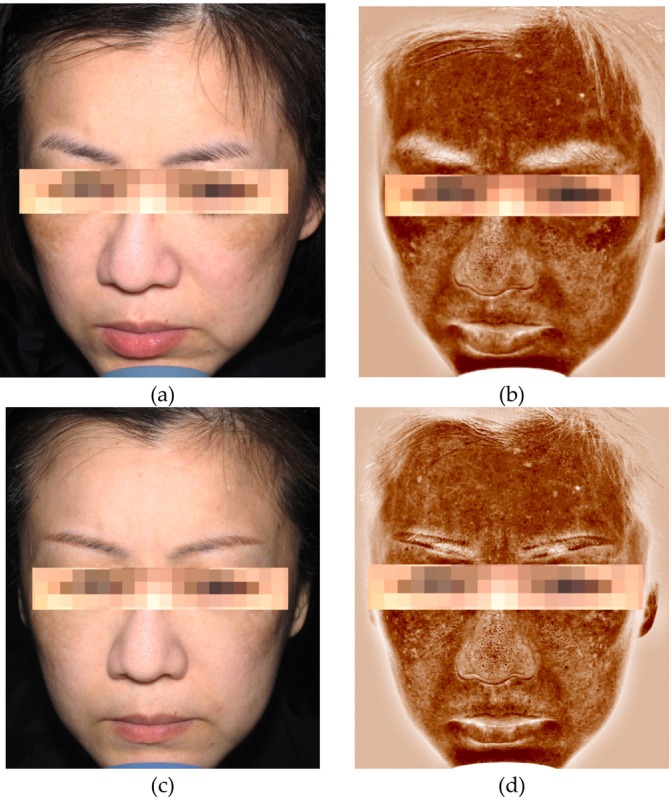
A decrease in pigmentation and melasma in **a** 40‐year‐old female patient (FST III) at baseline and after 5 treatment sessions. (a‐b) VISIA image before treatment; (c‐d) VISIA image after treatment.

A clinical case with a visible improvement after laser treatment are shown in Figure 7.

## 4 Discussion

The management of melasma remains a significant clinical challenge due to its chronic nature and the high risk of post‐inflammatory hyperpigmentation (PIH), particularly in patients with darker skin tones (Fitzpatrick types III‐V).

A central hurdle in melasma research is the lack of spatial reproducibility in follow‐up photography, as traditional “gold standard” tools like the MASI score are prone to inter‐observer variability and fail to capture localized chromatic shifts. To overcome this, our study employed a two‐stage alignment process based on SURF‐feature alignment and RANSAC, ensuring that identical midfacial Regions of Interest (ROIs) were analyzed with pixel‐perfect reproducibility.

By locking the coordinate system of the face, we eliminated positional noise and enabled an approach analogous to a “digital biopsy” of the lesion. This level of spatial precision is particularly fundamental when evaluating the Fractional Ruby Laser (694 nm); since this wavelength possesses the highest selectivity for melanin, its therapeutic impact often manifests as a complex spatial reorganization of pigment rather than a simple decrease in global intensity.

The most innovative aspect of our validation framework lies in its ability to capture subclinical pigmentary shifts through GLCM texture analysis. While conventional scales only record when a lesion appears lighter to the naked eye, GLCM tracks the fragmentation of melanin clusters, a phenomenon directly linked to the “shattering” effect of the Ruby laser on dermal pigment.

Our results suggest that the fractional ruby laser induces a transition toward textural uniformity. Even in cases where overall luminosity showed subtle changes, GLCM parameters identified the disruption of melanin‐laden macrophages and the breakdown of dense pigmentary aggregates. By providing an objective benchmark that captures treatment‐induced changes often missed by traditional scoring systems, this framework offers a granular view of how the laser alters the optical properties of the dermis, effectively mapping the transition from pathological clustering to a restored, homogenous skin texture.

Beyond direct pigmentary ablation, it is worth considering whether the efficacy of the 694 nm wavelength might also be partially linked to its potential impact on the vascular component of melasma. Melasma is increasingly hypothesized to be a multi‐factorial “photoaging” disorder in which dermal angiogenesis could play a significant role [14]. Supporting this view, diagnostic studies have reported that Vascular Endothelial Growth Factor (VEGF), a potent mediator of vascular endothelial cell proliferation and migration, is significantly upregulated in melasma lesional skin [15]. Crucially, the interaction between angiogenesis and pigmentation appears to be mediated by specific receptors; research has demonstrated that melanocytes express numerous VEGF receptors in vitro, including VEGFR‐1 and VEGFR‐2. The finding that VEGFR‐2 expression is upregulated after UV irradiation suggests a mechanism by which sun exposure simultaneously triggers both vascular expansion and melanocyte activation, potentially creating a pro‐melanogenic feedback loop [16].

In this context, our findings while primarily focused on textural and pigmentary changes do not exclude the possibility that the fractional Ruby laser might modulate the dermal microenvironment by tentatively disrupting this angiogenic‐melanogenic crosstalk.

While traditional vascular lasers like the Pulsed Dye Laser (PDL) and copper bromide (CuBr) are highly effective at targeting oxyhemoglobin, they often carry a significant risk of purpura or PIH in darker skin types [17, 18, 19]. In contrast, the 694 nm wavelength may offer a “dual‐target” potential: it penetrates deeply enough to reach the dilated dermal plexus, potentially reducing the chronic telangiectasia and the subsequent release of inflammatory mediators that drive melanocyte hyperactivity. This secondary vascular effect likely contributes to the reduction of the “muddy” erythematous‐pigmentary appearance of mixed melasma, providing a more stable clinical result.

Our findings align with and expand upon the existing literature, confirming that multiple sessions of low‐dose fractional Q‐switched Ruby Laser (QSRL) significantly reduce pigmentation while maintaining an excellent safety profile.

These results are consistent with the systematic review by Lee et al. (2022) [20], which emphasized that low‐fluence Q‐switched strategies specifically for the Nd:YAG laser minimize adverse events like mottled hypopigmentation. However, our study suggests that the 694 nm wavelength offers a strategic advantage; its higher absorption peak for melanin compared to the 1064 nm Nd:YAG allows for more precise targeting of chromophores with lower overall energy delivery, thereby reducing the cumulative thermal burden on the dermis.

The clinical efficacy of this approach was previously demonstrated by Jang et al. (2011) [3] in Korean patients, where six sessions led to a significant MASI reduction (from 15.1 ± 3.3 to 10.6 ± 3.9) and a notable increase in skin lightness (L‐value).

Our results further corroborate these findings, proving that substantial improvements in melasma can be achieved in Asian cohorts (Fitzpatrick III‐IV) with as few as three to five sessions [3]. This is particularly significant when compared to the work of Hilton et al. (2013) [4]; while their study reported high efficacy in Caucasian patients (Fitzpatrick I‐III), our data demonstrates equivalent safety in a population far more predisposed to Post‐Inflammatory Hyperpigmentation (PIH). The consistency of these results across different ethnicities highlights the robustness of the fractional approach: by creating Microscopic Thermal Zones (MTZs), we promote rapid epidermal repair and mitigate the stimulation of inflammatory mediators.

A critical factor in our observed safety profile was the implementation of a single‐pass protocol using a 9 mm High Coverage (HC) handpiece. Unlike traditional “laser toning” with 1064 nm Nd:YAG, which often requires thousands of shots and multiple passes potentially leading to cumulative thermal injury or guttate hypomelanosis our findings demonstrate that a single pass is sufficient to achieve therapeutic goals. This minimizes the risk of hyperpigmentation, which is particularly high for wavelengths with intense melanin absorption like the 694 nm or 532 nm. By reducing the number of passes, we decreased the cumulative thermal burden, effectively lowering the likelihood of PIH and shortening post‐treatment erythema [21].

The sensitivity of our ROI‐based digital analysis further elucidated these dynamics, revealing a statistically significant reduction in Type V skin color areas on the right side of the face (*p* = 0.036) that was not mirrored on the left (*p* = 0.235).

This discrepancy, likely linked to asymmetric UV exposure, underscores the ability of our framework to detect localized variations that global scales often average out. Ultimately, the integration of fractional 694 nm technology with an objective imaging framework moves melasma management away from subjective evaluation toward a reproducible, data‐driven science, offering a promising approach for the long‐term management of this challenging condition even in high‐risk skin phototypes.

Despite the positive outcomes, the retrospective nature of this study and the six‐month follow‐up period limit insights into long‐term recurrence rates. However, the integration of fractional 694 nm technology with an AI‐assisted quantitative imaging framework moves melasma management away from subjective evaluation toward a reproducible, data‐driven science. This methodology not only validates the ruby laser's performance but also provides a more sensitive tool for the early detection of therapeutic response, ultimately offering a promising approach for the long‐term management of this challenging condition.

## 5 Conclusion

This study confirms that a low‐fluence fractional 694‐nm Q‐switched Ruby laser protocol is a safe and highly effective treatment for melasma in Asian patients with Fitzpatrick skin types III–V. By targeting melanin with high specificity and utilizing a fractional delivery system, we achieved significant clearing of hyperpigmented lesions while maintaining an excellent safety profile, notably avoiding common complications such as post‐inflammatory hyperpigmentation or leukoderma.

Beyond clinical outcomes, our work highlights the critical role of AI‐assisted imaging in modern dermatology. The transition from subjective visual scales to an objective framework—integrating SURF‐RANSAC alignment and GLCM texture analysis—allows for the detection of subclinical melanin fragmentation and textural shifts. This “digital biopsy” approach provides a granular benchmark for therapeutic response that traditional scoring systems fail to capture. Ultimately, the synergy between advanced laser technology and objective digital validation offers a more precise, reproducible, and data‐driven path for the long‐term management of melasma.

## Author Contributions

**Patrick Po‐Han Huang:** conceptualization, methodology, supervision, writing, data curation. **Ilia Tetin:** conceptualization, methodology, writing. **Chih‐Yu Wang:** conceptualization, methodology. **Sherry Yunshan OuYang:** investigation. **Pei‐Ling Chih:** investigation. **Daniela Martinelli, Alessandra Zevini** and **Riccardo Barini:** data analysis, supervision, writing – review and editing. All authors approved the final version of the manuscript.

## Ethics Statement

Approval by the Mackay Memorial Hospital Institutional Review Board, pursuant to Protocol No.: 21MMHIS317e dated May 19, 2022, issued by the Taiwan Christian Foundation for the Elderly, Mackay Memorial Hospital Institutional Review Board. The study was conducted in accordance with the Declaration of Helsinki. The device has been CE Marked since 2014 and registered for Taiwan market since 2017.

## Consent

All participants in the study provided their informed consent to the publication of their images.

## Conflicts of Interest

Daniela Martinelli, Alessandra Zevini, and Riccardo Barini are employed at El.En. Group. The other authors declare that the research was conducted in the absence of any commercial or financial relationships that could be construed as potential conflicts of interest.

## Data Availability

The data that support the findings of this study are available from the corresponding author upon reasonable request.
